# Evaluation of the use of Global Youth Tobacco Survey (GYTS) data for developing evidence-based tobacco control policies in Turkey

**DOI:** 10.1186/1471-2458-8-S1-S4

**Published:** 2008-12-15

**Authors:** Toker Erguder, Banu Çakır, Dilek Aslan, Charles W Warren, Nathan R Jones, Samira Asma

**Affiliations:** 1 World Health Organization Country Office, Birlik Mahallesi 2. Cadde No: 11, Cankaya, Ankara, 06610 Turkey; 2 Department of Public Health, Hacettepe University Faculty of Medicine, Sihhiye, Ankara, 06100 Turkey; 3 Office on Smoking and Health, Centers for Disease Control and Prevention, 4770 Buford Highway NE Mailstop K-50, Atlanta, Georgia, 33041, USA

## Abstract

**Introduction:**

The tobacco control effort in Turkey has made significant progress in recent years. Turkey initiated its tobacco control effort with the passing of Law 4207 (The Prevention of Harmful Effects of Tobacco Products) in 1996 and ratified the World Health Organization (WHO) Framework Convention on Tobacco Control (FCTC) in 2004. It is important to base policy decisions on valid and reliable evidence from population-based, representative studies that are periodically repeated to enable policy makers to monitor the results of their interventions and to appropriately tailor anti-tobacco activities towards future needs.

**Methods:**

The Global Youth Tobacco Survey (GYTS) was developed to track tobacco use among young people and enhance the capacity of countries to design, implement, and evaluate tobacco control and prevention programs. Turkey conducted the GYTS in 2003 and data from this survey can be used as baseline measures for evaluation of the tobacco control programs implemented by the Ministry of Health (MOH) of the Turkish government.

**Results:**

The GYTS was conducted in 2003 on a representative sample of students aged 13 to 15 years. It indicated that almost 3 in 10 students in Turkey had ever smoked cigarettes, with significantly higher rates among boys. Current cigarette smoking rates were lower, at 9% for boys and 4% for girls. The prevalence of current use of other tobacco products was about half these figures for each gender. About 80% were exposed to secondhand smoke. Exposure to pro-smoking media messages was not rare. Almost half of the smokers 'usually' bought their tobacco from a store, despite the law prohibiting this. Exposure to teaching against smoking in schools was not universal.

**Conclusion:**

Findings from the GYTS, with periodic repeats of the survey, can be used to monitor the impact of enforcing various provisions of the present law (No: 4207), the progress made in achieving the goals of the WHO FCTC, and the effectiveness of various preventive interventions against smoking. Such data would inform and help in the development of public health strategy.

## Introduction

The tobacco control effort in Turkey has made significant progress in recent years. In December 2004, Turkey ratified the World Health Organization (WHO) Framework Convention on Tobacco Control (FCTC) [1,2]. The WHO FCTC encourages countries to develop and implement national tobacco control action plans, including public policies such as: bans on direct and indirect tobacco advertising; raises in tobacco tax and price; promotion of smoke-free public places and workplaces; and provision of health messages on tobacco packaging. Turkey has exerted a great effort to meet the requirements of the WHO FCTC in a timely manner.

In 2005, the Ministry of Health (MOH) formed a working group, including participants from non-governmental organizations, universities, and other relevant institutions, to develop goals and objectives for a National Tobacco Control Program (NTCP) for Turkey. With regard to the prevalence of tobacco use, the working group produced two main objectives: to reduce the prevalence of tobacco use by adults (aged 15 years and over) to 20% by the year 2010; and to reduce the prevalence of tobacco use by youths (<15 years of age) to zero by 2010.

The government of Turkey approved the implementation of the NTCP on December 27, 2007. In addition, as recommended by the working group, the MOH has presented to the National Grand Assembly some amendments to Law 4207 (The Prevention of Harmful Effects of Tobacco Products) [3]. Law 4207 initially included provisions banning advertising of tobacco products, sale of tobacco products to minors, and smoking in public places. The proposed amendments to Law 4207 include additional provisions, such as: extending the scope of restrictions on consumption of tobacco and tobacco products in public areas; prohibiting the sale of tobacco and tobacco products through vending machines; and prohibiting the appearance of smoking in all audio-visual media.

The MOH has taken leadership in several efforts to prevent tobacco use and promote cessation activities, such as: sponsoring anti-tobacco television and radio programs and seminars; conducting public education and anti-tobacco activities utilizing posters, brochures and leaflets; training health professionals on patient counseling regarding tobacco use; establishing cessation centers; and continuing the 'Quit and Win Campaign'. (The Quit and Win campaign, an international smoking cessation competition to motivate smokers to quit smoking, was first started in Turkey in 1996 and has been carried out by the Substance Dependence Section, Mental Health Department, General Directorate of Primary Health Care, Ministry of Health, Turkey under the auspices of the WHO every second year.)

In addition to the tobacco control program efforts, one important feature of the WHO FCTC is the call for countries to establish programs for national, regional, and global surveillance [1]. WHO, the US Centers for Disease Control and Prevention, and the Canadian Public Health Association developed the Global Tobacco Surveillance System (GTSS) to assist WHO member states in establishing continuous periodic tobacco control surveillance and monitoring [4,5]. The GTSS includes collection of data through three surveys: the Global Youth Tobacco Survey (GYTS) for youth, and the Global School Personnel Survey and the Global Health Professionals Survey for adults [4].

The purpose of this paper is to present some data from 13- to 15-year-old students who completed the GYTS conducted in Turkey in 2003 [6,7] and to scrutinize the findings of the GYTS that can be used to monitor provision of the tobacco control laws in Turkey, as well as the articles in the WHO FCTC.

## Methods

### The Global Youth Tobacco Survey (GYTS)

The GYTS uses a standardized methodology for constructing sampling frames, selecting schools and classes, preparing the questionnaire, carrying out field procedures, and processing data. The GYTS questionnaire is self-administered in classrooms, and school, class, and student anonymity is maintained throughout the GYTS process. Country-specific questionnaires consist of a core set of questions that all countries ask, as well as unique country-specific questions. The final country questionnaires are translated to local languages, and back-translated to check for accuracy. GYTS country research coordinators conduct focus group discussions with students aged 13 to 15 years to further test the accuracy of the translation and students' understanding of the questions.

The GYTS enquired about several important tobacco-use indicators, including: current cigarette smoking (based on a response of "1 or more days" to the question, "During the past 30 days (1 month), on how many days did you smoke cigarettes?"); current use of tobacco products other than cigarettes; 'susceptibility' (that is, absence of a firm decision not to smoke) or likely initiation of cigarette smoking in the next year among never smokers (based on a negative response to the question, "Have you ever tried or experimented with cigarette smoking, even one or two puffs?" as well as a response of anything but "definitely no" to the questions, "If one of your best friends offered you a cigarette, would you smoke it?" and "Do you think you will try smoking a cigarette in the next year?") [8]; exposure to cigarette smoke in public places (based on a response of "1 or more days" to the question, "During the past 7 days, on how many days have people smoked in your presence, in places other than your home?"); one or more parents smoke cigarettes (based on a response of "both", "father only", or "mother only" to the question, "Do your parents smoke?"); one or more best friends smoke cigarettes (based on a response of "most" or "all" to the question, "Do most or all of your best friends smoke?"); in favor of banning cigarette smoking in public places (based on a positive response to the question, "Are you in favor of banning smoking in public places (such as in restaurants, in buses, streetcars, and trains, in schools, on playgrounds, in gyms and sports arenas, in discos?"); and exposure to pro-tobacco advertising and promotion, either direct or indirect (based on: a response of "a lot" or "a few" to the questions, "During the past 30 days (1 month), how many anti-smoking media messages (for example, television, radio, billboards, posters, newspapers, magazines, movies, drama) have you seen or heard?", "During the past 30 days (1 month), how many advertisements for cigarettes have you seen on billboards?", "During the past 30 days (1 month), how many advertisements for cigarettes have you seen at point of sale?", "During the past 30 days (1 month), how many advertisements or promotions for cigarettes have you seen in newspapers or magazines?"; a positive response to the questions, "Do you have something (t-shirt, pen backpack, etc) with a cigarette brand logo on it?" or "Has a cigarette company representative ever offered you a free cigarette?").

The GYTS data in this report include a representative national estimate, compiled from 10 separate surveys conducted in Turkey in 2003: three major cities (Ankara, Istanbul and Izmir) and seven geographical regions (Aegean, Marmara, Black Sea, Mediterranean, Inner Anatolia, Eastern Anatolia, and South-Eastern Anatolia). In total, 15,957 students completed the GYTS. The GYTS sample design produces representative, independent, cross-sectional estimates for each site. A weighting factor was applied to each student record to adjust for the probability of selection at the school, class, and student levels, and non-response at the school, class, and student levels. A final adjustment sums the weights by grade and gender to the population of school children in the selected grades in each sample site. Thus, the national data are a compilation of the individual site data. SUDAAN, a software package for statistical analysis of correlated data, was used to compute standard errors of the estimates and produced 95% confidence intervals, which are shown in the manuscript as lower and upper bounds [9]. *t*-Tests were used to determine differences between subpopulations [10]. Statistical differences are noted at the *p *< 0.05 level. Given that gender is an effect modifier for the association between smoking and various risk factors [11,12], all statistical analyses were conducted stratifying on gender. This paper is limited to students aged 13 to 15 years old.

The findings in this report are subject to at least three limitations. First, because the sample surveyed was limited to youths attending school, they may not be representative of all 13 to 15 year olds in Turkey. Second, these data apply only to youths who were in school the day the survey was administered and completed the survey. The school response rate was 100% in all sites. The student response rate was over 90% in all sites, except South-Eastern Anatolia (84.0%), suggesting bias due to absence or non-response is small. Third, data are based on self reports of students, who may under- or over-report their use of tobacco. The extent of this bias cannot be determined in this particular group; however, responses to tobacco questions on surveys similar to GYTS have shown good test-retest reliability [13].

## Results

### Prevalence

Almost 3 in 10 (26.3%) students in Turkey ever smoked cigarettes, with the rate for boys (31.7%) significantly higher than for girls (19.7%) (Table 1). Of ever smokers in Turkey, 30.7% initiated smoking before age 10, with a significantly higher rate among boys (34.9%) compared to girls (23.7%). Overall, 6.9% of students in Turkey currently smoked cigarettes, with again a significantly higher rate among boys (9.4%) compared to girls (3.5%). Overall, 3.4% of students currently used tobacco products other than cigarettes; the rate for boys (4.4%) was about 3 times the rate for girls (1.5%). Slightly over 1 in 10 (13.1%) students who currently smoked cigarettes reported they "feel like having a cigarette first thing in the morning" (suggesting tobacco dependency). Among never smoker adolescents aged 13 to 15 years, 7.0% indicated that they were susceptible to initiate smoking during the next year (8.2% of boys and 5.3% of girls).

**Table 1:** Prevalence of tobacco use and interest in stopping smoking among students aged 13 to 15 years by gender, Turkey Global Youth Tobacco Survey, 2003

	**Prevalence % (95% CI)**		
	
	**Total**	**Male**	**Female**
**Prevalence**			
Ever smoked cigarettes, even one or two puffs	26.3 (24.3–28.4) (n = 11,150)	31.7 (29.0–34.5) (n = 5,621)	19.7 (17.6–22.0) (n = 5,275)
Ever smokers who initiated smoking before age 10	30.7 (28.0–33.4) (n = 2,677)	34.9 (32.4–37.4) (n = 1,637)	23.7 (19.5–28.5) (n = 942)
Current cigarette smokers	6.9 (6.1–7.9) (n = 10,949)	9.4 (8.2–10.9) (n = 5,520)	3.5 (2.9–4.3) (n = 5,188)
Currently use of other tobacco products	3.4 (3.0–3.9) (n = 11,290)	4.4 (3.8–5.1) (n = 5,731)	1.5 (1.1–1.9) (n = 5,285)
Current cigarette smokers who felt like having a cigarette first thing in the morning	13.1 (9.6–17.6) (n = 347)	12.8 (9.0–17.8) (n = 229)	13.6 (7.2–24.3) (n = 86)
Never smokers likely to initiate smoking in the next year	13.1 (9.6–17.6) (n = 347)	12.8 (9.0–17.8) (n = 229)	13.6 (7.2–24.3) (n = 86)
	13.1 (9.6–17.6) (n = 347)	12.8 (9.0–17.8) (n = 229)	13.6 (7.2–24.3) (n = 86)
**Cessation**			
Current cigarette smokers wanting to stop smoking	65.3 (60.4–69.9) (n = 410)	68.9 (63.0–74.3) (n = 295)	60.2 (50.6–69.1) (n = 88)
Current cigarette smokers who had tried to stop smoking during the past year	61.4 (55.7–66.8) (n = 439)	66.9 (60.5–72.7) (n = 316)	44.8 (31.9–58.4) (n = 90)
Current smokers who had ever received help to stop smoking	71.5 (67.0–75.5) (n = 622)	75.3 (71.3–79.0) (n = 436)	55.6 (44.5–66.2) (n = 147)

CI, confidence interval.

### Cessation

Among students who currently smoked cigarettes in Turkey, over 6 in 10 students (65.3%) reported that they "want to stop smoking now"; 61.4% stated that they "tried to stop smoking during the past year but failed"; and 71.5% reported that they "had received help to stop smoking" (Table 1). Among current smokers, boys were significantly more likely than girls to have tried to stop smoking or to have ever received help to stop smoking.

### Exposure to secondhand smoke

Over 8 in 10 students in Turkey reported that they were exposed to smoke from others in their home (81.6%) and in public places (85.9%) (Table 2). Over 9 in 10 (91.4%) students in Turkey thought "smoking should be banned in public places"; support rates were significantly higher among girls (94.0%) than among boys (89.6%).

**Table 2:** Prevalence of factors influencing tobacco use among students aged 13 to 15 years by gender, Turkey Global Youth Tobacco Survey, 2003

	**Prevalence % (95% CI)**		
	
	**Total**	**Male**	**Female**
**Secondhand smoke exposure**			
Exposure to smoking from others at home in the past 7 days	81.6 (80.6–82.5) (n = 11,197)	80.6 (79.4–81.7) (n = 5,643)	82.7 (81.4–83.8) (n = 5,300)
Exposure to smoke in public places in the past 7 days	85.9 (84.8–87.0) (n = 11,205)	85.3 (83.8–86.6) (n = 5,696)	86.9 (85.7–87.9) (n = 5,274)
Those who thought smoking should be banned in public places	91.4 (90.6–92.1) (n = 11,323)	89.6 (88.5–90.6) (n = 5,733)	94.0 (93.0–94.9) (n = 5,335)
**Pro-tobacco advertising exposure**			
Exposure to advertisements for cigarettes on billboards in the past month	33.4 (32.1–34.8) (n = 10,764)	34.4 (32.8–36.0) (n = 5,429)	32.1 (30.4–33.8) (n = 5,109)
Exposure to advertisements for cigarettes in newspapers or magazines in the past month	27.9 (26.8–29.1) (n = 10,984)	29.9 (28.1–31.7) (n = 5,529)	25.1 (23.7–26.5) (n = 5,225)
Ownership of an object with a tobacco brand logo on it	10.1 (9.3–11.0) (n = 11,129)	12.4 (11.4–13.5) (n = 5,634)	6.7 (5.8–7.7) (n = 5,268)
**Access and availability of cigarettes to smokers**			
Current smokers who usually bought their cigarettes in a store	46.5 (40.6–52.4) (n = 557)	48.9 (41.8–55.9) (n = 387)	38.4 (28.7–49.3) (n = 129)
Current smokers who usually bought their cigarettes in a store who had never been refused purchase because of their young age	86.4 (80.5–90.8) (n = 226)	88.0 (81.2–92.6) (n = 166)	82.7 (66.6–92.0) (n = 39)
Those ever been offered "free" cigarettes by a cigarette company representative	7.6 (7.0–8.2) (n = 10,530)	9.1 (8.2–10.1) (n = 5,262)	5.6 (4.9–6.3) (n = 5,093)
**Exposure to teaching in school the previous year about:**			
Dangers of smoking tobacco	52.8 (50.9–54.7) (n = 11,051)	52.7 (50.7–54.6) (n = 5,609)	53.3 (50.9–55.7) (n = 5,224)
Reasons why people their age smoke	21.1 (19.7–22.5) (n = 11,185)	21.3 (19.8–22.7) (n = 5,665)	20.5 (18.5–22.7) (n = 5,284)
The effects of smoking	40.1 (38.2–41.9) (n = 11,110)	39.4 (37.4–41.4) (n = 5,617)	41.4 (39.1–43.8) (n = 5,265)

CI, confidence interval.

### Media and advertising exposure

Over 3 in 10 (33.4%) students in Turkey reported that they saw advertisements for cigarettes on billboards during the month prior to the survey. Approximately 3 in 10 (27.9%) students reported that they saw advertisements for cigarettes in newspapers or magazines in the month prior to the survey. Boys (29.9%) were significantly more likely than girls (25.1%) to be exposed to pro-tobacco advertising in newspapers and magazines. Approximately 1 in 10 (10.1%) students reported that they had an object (that is, hat, t-shirt, pen, backpack, and so on) with a cigarette brand logo on it. Boys (12.4%) were significantly more likely than girls (6.7%) to own such an object.

### Access and availability

Almost half (46.5%) of current smokers reported that they "usually" bought their tobacco from a store (Table 2). Current smokers who usually bought their cigarettes from a store were asked if they had ever been refused purchase because of their young age: almost 9 in 10 (86.4%) reported they had not been refused anytime. It is important that all study participants were 15 years old or younger (that is, minors) and, thus, should not have been allowed by law to purchase tobacco.

Students were asked if they had been offered "free" cigarettes by a tobacco company representative at any time. Overall, 7.6% of students had been offered "free" cigarettes, with a significantly higher rate for boys (9.1% versus 5.6%).

### School curricula

Students were asked if, during the past school year in classes, they had been taught about the dangers of tobacco, discussed the reasons why young people smoke, or if they had been taught about the effects of tobacco on their health. Half (52.8%) of the students reported that they had been taught about the dangers of tobacco; only 21.1% had discussed the reasons why young people use tobacco; and 40.1% had been taught about the effects of tobacco on their health (Table 2).

## Discussion

Tobacco-related studies are quite common in Turkey, yet most are either small-scale or not representative of the entire population. Besides prevalence studies in different population sub-groups [14], there have been a number of studies on potential risk factors for smoking [12] and/or on assessment and monitoring of tobacco control studies in Turkey [15]. On the other hand, there is still a missing link between such evidence and tobacco-related policy making. It is important to base policy decisions on valid and reliable evidence from population-based, representative studies that are periodically repeated to enable policy makers to monitor the results of their interventions and to appropriately tailor anti-tobacco activities to future needs.

The WHO FCTC calls for countries to use consistent methods and procedures in their surveillance efforts. The GYTS was designed for exactly this purpose (that is, standardized sampling procedures, core questionnaire items, training in field procedures, and analysis of data, all of which are consistent across all survey sites). The GYTS provides indicators for measuring achievement of five WHO FCTC articles (namely, exposure to secondhand smoke, school-based tobacco control curricula, cessation, media and advertising, and minors' access and availability to tobacco products) and results from the 2003 GYTS can be used to set baseline measures that can be used to monitor the five WHO FCTC articles, as discussed below.

### Article 8: protection from exposure to tobacco smoke

Law 4207 calls for a ban on smoking in public places; however, the ban is limited to health care facilities, schools, theatres and cinemas, and some public transport (buses, trains, and domestic and international air transport) [3]. Results of the GYTS (2003) suggest that the vast majority of Turkish teenagers are exposed to secondhand smoke in public places. The MOH is now focusing on developing a proposal to amend Law 4207 to include provisions to prohibit the consumption of tobacco and tobacco products in additional public places. To assist in this effort, the GYTS data show that over 94% of never smokers and 64.1% of current smokers are in support of banning smoking in public places, with no significant difference by gender [6]. In a similar way, 81.6% of never smokers (slightly higher in girls versus boys) and 55.3% of current smokers (with no significant difference by gender) report that they believe passive smoking is harmful [6].

### Article 12: education, communication, training and public awareness

From the GYTS data, half of the students in Turkey reported that, during the past school year, they had been taught about the dangers of smoking and one-third had discussed reasons why people their age smoke. This information calls for the development, implementation and evaluation of evidence-based tobacco prevention curricula to be used in schools. The centralized education system in Turkey will facilitate national implementation of a tobacco use prevention curriculum, once an effective program is identified and evaluated.

It is important to note that introducing anti-smoking programs to teenagers is not an easy task. It is not always easy to identify the smoking students, given that they do not want this to be known by their teachers, parents, and other relatives and/or may be scared that they will be punished for smoking. Similarly, because of a fear of stigmatization, for example, teenage smokers are not as open as adults to attending 'quit smoking' centers and/or to receiving help from professionals, even when they want to quit smoking. Training for teachers and parents in how to approach a teenager who smokes is important in this respect.

### Article 13: tobacco advertising, promotion and sponsorship

Law 4207 prohibits mass media advertising of cigarettes and other tobacco products, as well as the sponsorship of all sports and cultural events by tobacco companies. Data from the GYTS show one-third of the students reported seeing cigarette advertisements on billboards and 28% saw advertisements for cigarettes in newspapers or magazines. In other countries that have conducted the GYTS, billboards often refer to posters seen at point of sale locations and newspapers and magazines refer to international publications. Turkey does not have a ban on advertisements in international publications.

The GYTS also includes information on indirect advertising by the tobacco industry. Students were asked if they have an object (cap, shirt, knapsack, and so on) with a tobacco company logo on it. One in 10 students in Turkey had such an object. The working group formed by the MOH could use this information as support for amending Law 4207 to include stronger provisions regarding direct and indirect advertising of tobacco products.

### Article 14: demand reduction measures concerning tobacco dependence and cessation

The GYTS results show 7 in 10 current smokers wanted to stop smoking and over 7 in 10 have tried to stop during the past year but have failed. Turkey has some cessation clinics in select universities and hospitals, but these cessation efforts focus largely on adults. This finding indicates a need to develop, pilot test, and evaluate potential youth cessation programs. Once effective programs have been identified, they need to be made widely available throughout Turkey. The evidence is currently insufficient to support the efficacy of smoking cessation programs in young people [16].

### Article 16: sales to and by minors

The GYTS data show that almost half of the current smokers (all aged 13 to 15 years and, thus, minors) usually buy their cigarettes in a shop and many of them were not refused purchase because of their age. Clearly, enforcement of the minors' access law is a major issue facing Turkey, as well as amendment of the prohibition of tobacco sale near educational facilities. These efforts can complement other population-based intervention efforts to reduce tobacco use in Turkey.

## Conclusion

Passing of Law 4207, ratification of the WHO FCTC, and formation of a working group by the MOH to assist in developing a national tobacco control program are important milestones for tobacco control in Turkey. The MOH now needs to move forward in implementing the provisions of Law 4207, and developing effective enforcement procedures. The working group and the MOH can use the findings of the GYTS to assist in development of a national tobacco control policy, as recommended in the WHO European Strategy for Tobacco Control [17]. Development of an effective comprehensive NTCP-including smoke-free environment policies, increases in the price of tobacco products, comprehensive laws to regulate and enforce bans on sales, purchases, and consumption of tobacco products by underage youth, regulations of content, labeling, promotion, and advertising of tobacco products, and targeted mass media campaigns [18,19] – will require careful monitoring and evaluation of the existing programs and should be tailored to future needs. The synergy between Turkey's leadership in passing Law 4207, ratifying the WHO FCTC, and supporting the conduct of the GYTS offers Turkey a unique opportunity to develop, implement and evaluate a comprehensive tobacco control policy that can be most helpful to Turkey.

## List of abbreviations used

FCTC: Framework Convention on Tobacco Control; GTSS: Global Tobacco Surveillance System; GYTS: Global Youth Tobacco Survey; MOH: Ministry of Health; NTCP: National Tobacco Control Program; WHO: World Health Organization.

## Competing interests

The authors declare that they have no competing interests.

## Authors' contributions

CWW, NRJ and SA conceived the study, participated in its design and coordination, and helped to draft the manuscript. TE was responsible for national coordination of the study and for drafting the manuscript. BC and DA participated in interpretation of the statistical analysis and drafting the manuscript. All authors read and approved the final manuscript.
